# Depletion of ASK1 blunts stress-induced senescence in adipocytes

**DOI:** 10.1080/21623945.2020.1815977

**Published:** 2020-09-15

**Authors:** Stephan Wueest, Fabrizio C. Lucchini, Yulia Haim, Assaf Rudich, Daniel Konrad

**Affiliations:** a Division of Pediatric Endocrinology and Diabetology, University Children’s Hospital, Zurich, Switzerland; b Children’s Research Center, University Children’s Hospital, Zurich, Switzerland; c Zurich Center for Integrative Human Physiology, University of Zurich, Zurich, Switzerland; d Department of Clinical Biochemistry and Pharmacology, Ben-Gurion University of the Negev, Beer-Sheva, Israel; e The National Institute of Biotechnology in the Negev, Ben-Gurion University of the Negev, Beer-Sheva, Israel

**Keywords:** Obesity, diabetes, browning, adipose tissue, subcutaneous, lipopolysaccharide, p38 MAPK

## Abstract

Increasing energy expenditure via induction of browning in white adipose tissue has emerged as a potential strategy to treat obesity and associated metabolic complications. We previously reported that ASK1 inhibition in adipocytes protected from high-fat diet (HFD) or lipopolysaccharide (LPS)-mediated downregulation of UCP1 both *in vitro* and *in vivo*. Conversely, adipocyte-specific ASK1 overexpression attenuated cold-induction of UCP-1 in inguinal fat. Herein, we provide evidence that both TNFα-mediated and HFD-induced activation of p38 MAPK in white adipocytes are ASK1-dependent. Moreover, expression of senescence markers was reduced in HFD-fed adipocyte-specific ASK1 knockout mice. Similarly, LPS-induced upregulation of senescence markers was blunted in ASK1-depleted adipocytes. Thus, our study identifies a previously unknown role for ASK1 in the induction of stress-induced senescence.

## Introduction

The prevalence of obesity and its associated co-morbidities has reached pandemic proportions. Dissipation of energy in brown and/or beige adipocytes has emerged as a promising target to combat obesity. It depends on uncoupling protein 1 (UCP1)-mediated proton leak across the inner mitochondrial membrane leading to heat production and other energy-expending ‘futile’ cycles such as creatine and calcium cycling [1]. Since adult humans have little or no existing active brown adipose tissue (BAT), selective induction of white AT (WAT) beiging/browning may have the greater therapeutic potential to fight obesity [2,3]. We recently demonstrated a role for the apoptosis signal-regulating kinase 1 (ASK1) in mediating the inhibitory effect of caloric surplus or LPS-treatment on adipose tissue browning but not on brown adipose tissue activation [4]. Particularly, high-fat diet (HFD)-fed adipocyte-specific ASK1 knockout (ASK1^Δadipo^) mice revealed increased UCP1 protein levels in inguinal adipose tissue concomitant with elevated energy expenditure, reduced obesity and ameliorated glucose tolerance compared to control littermates. Moreover, lipopolysaccharide (LPS)-mediated downregulation of isoproterenol-induced UCP1 was blunted in subcutaneous fat of ASK1^Δadipo^ mice. Conversely, adipocyte-specific ASK1 overexpression in chow-fed mice attenuated cold-induced UCP-1 protein levels in inguinal fat.

Cold temperatures induce WAT browning. However, the potential to form cold-induced beige adipocytes in subcutaneous WAT is lost with age [5]. The latter is due to a senescence-like phenotype of beige progenitor cells through activation of the p38 MAPK-p16Ink4a pathway [6]. Within WAT, senescence not only occurs in dividing cells such as progenitors but also in non-dividing adipocytes [7]. Indeed, cultured white adipocytes treated with serum harvested from old mice revealed elevated expression of the senescence markers *p16^Ink4a^* and *p21* compared to cells treated with serum from young mice, indicating that ageing induces senescence in adipocytes [8]. Importantly, p38 MAPK is a mitogen-activated protein kinase that is activated downstream of ASK1 [9]. The latter acts as a signalling node in which different stressors such as endoplasmic reticulum, oxidative and inflammatory stresses converge [10]. In fact, the pro-inflammatory cytokine tumour necrosis factor-alpha (TNFα) and the endotoxin LPS, which are both elevated in obesity [11,12], are known activators of ASK1 [10,13]. Herein, we aimed to analyse whether increased UCP1 expression in ASK1-depleted inguinal adipose tissue [4] is paralleled by an altered senescence status. We hypothesized that activation of the p38 MAPK-senescence pathway in subcutaneous adipocytes is ASK1-dependent.

## Materials and methods

### Animals

To obtain adipocyte-specific ASK1 depletion (ASK1^Δadipo^), homozygous ASK1 floxed mice were bred to mice expressing Cre under the adiponectin promoter (AdipoqCre) as previously reported [4,14]. Littermate mice with floxed ASK1 but absent Cre-recombinase (Cre) expression were used as controls (ASK1^F/F^). Male mice were housed in a specific pathogen-free environment on a 12 h-light-dark cycle (light on from 7 am to 7 pm) and fed ad libitum with high-fat diet (HFD) (58 kcal% fat w/sucrose Surwit Diet, D12331, Research Diets). HFD was started at an age of 6 weeks. All protocols conformed to the Swiss animal protection laws and were approved by the Cantonal Veterinary Office in Zurich, Switzerland.

### Western blotting

Tissues or cells were lysed in ice-cold lysis buffer containing 150 mM NaCl, 50 mM Tris-HCl (pH 7.5), 1 mM EGTA, 1% NP-40, 0.25% sodium deoxycholate, 1 mM sodium pyrophosphate 1 mM sodium vanadate, 1 mM NaF, 10 mM sodium β-glycerolphosphate, 0.2 mM PMSF and a 1:1000 dilution of protease inhibitor cocktail (Sigma-Aldrich, Saint-Louis, MI, USA). Protein concentration was determined using a BCA assay (Pierce, Rockford, IL, USA). Equal amounts of protein were resolved by LDS-PAGE (4–12% gel; NuPAGE, Invitrogen, Basel, Switzerland) and electro-transferred onto nitrocellulose membranes (0.2 μm, BioRad, Reinach, Switzerland). Equal protein loading on membranes was checked by Ponceau S staining. Blots were blocked in tris-buffered saline (50 mM Tris-HCl, 150 mM NaCl) containing 0.1% Tween (TBS-T) supplemented with 5% non-fat dry milk. Membranes were then placed in a 50 ml Falcon tube and incubated overnight at 4°C with gentle rotation with the respective primary antibody solutions. Antibody-antigen complexes were detected by using the ECL system and detected with the Fuji LAS-3000 image reader (Fujifilm, Tokyo, Japan). The following primary antibodies (diluted 1:1000) were used Actin, MAB1501 (Millipore, Darmstadt, Germany); p53, #2524, p38, #9212 and phospho-p38, #9211, HSP90, #4877 (Cell Signalling, Danvers, MA, USA), p21, ab109199 (Abcam, Cambridge, UK).

### RNA extraction and quantitative reverse transcription-PCR (RT-PCR)

Total RNA was extracted with NucleoSpin® RNA kits (Macherey-Nagel, Düren, Germany). RNA concentration was determined spectrophotometrically using NanoDrop® (ThermoFisher Scientific, Waltham, MA, USA). RNA was reverse transcribed with PrimeScript RT reagent kit using random hexamer primers (Takara, Kusatsu, Japan). TaqMan system was used for real-time PCR amplification. Relative gene expression was obtained after normalization to 18s RNA, using the formula 2^−ΔΔcp^. The following primers/probes were used *Il-6* Mm00446190_m1, *Il-8* Mm00433859_m1, *p16^Ink4a^* Mm00494449_m1 and *p21* Mm00432448_m1 (Applied Biosystems, Rotkreuz, Switzerland).

### MEFs

MEFs harvested from ASK1^F/F^ or ASK1^Δadipo^ mice were differentiated as described [15]. Differentiated adipocyte-like MEFs were treated with 10 ng/ml recombinant TNFα for 30 min.

### Experiments in mature subcutaneous adipocytes

Generation and characterization of immortalized subcutaneous white pre-adipocytes were described previously [16]. Cells were seeded onto collagen-coated plates and grown in Dulbecco’s modified Eagle’s medium (DMEM) containing 25 mM glucose supplemented with 10% foetal bovine serum (FBS) and 1% penicillin/streptomycin (all from Invitrogen, Basel, Switzerland) (complete medium, CM) prior to differentiation. Two days post-confluent, subcutaneous white pre-adipocytes cells were treated with a mixture of methylisobutylxanthine (500 μM), dexamethasone (1 μM), insulin (1.7 μM) and rosiglitazone (1 μM) in CM to induce differentiation. Two days later, medium was changed to high glucose culture medium-containing insulin (0.5 μM). Subsequently, culture medium was replaced every other day. Seven days after induction of differentiation, mature scWAT adipocytes were reverse transduced with shLuc or shASK1 (12.5 MOI) lentiviral particles in CM supplemented with polybrene (8 μg/ml). Lentivirus was produced as described [4]. After 6 h, medium was replaced to CM supplemented with 0.5 μM insulin and changed every other day until the day of experiment. Eleven days after induction of differentiation, cells were treated with 100 ng/ml LPS from Escherichia (coli 055: B5, Sigma-Aldrich, Saint-Louis, MI, USA) for 24 h. The next day, cells were treated with 0.1 µM isoproterenol (Sigma-Aldrich) for 6 h. Subsequently, cells were immediately frozen at −80°C until further processing.

### Data analysis

Data are presented as means ± SEM. To assess normal distribution, D’Agostino-Pearson (n ≥ 8) or Shapiro–Wilk test (n < 8) was used. When comparing two groups, Mann–Whitney test was used for not normally and unpaired two-tailed Student’s *t* test for normally distributed data. When comparing more than two groups, two-way ANOVA with Tukey’s multiple comparison test was used. All statistical tests were calculated using the GraphPad Prism 8.00 (GraphPad Software, San Diego, CA, USA). P values < 0.05 were considered to be statistically significant.

## Results

### P38 MAPK is activated ASK1-dependently in adipocytes

**Figure 1.**
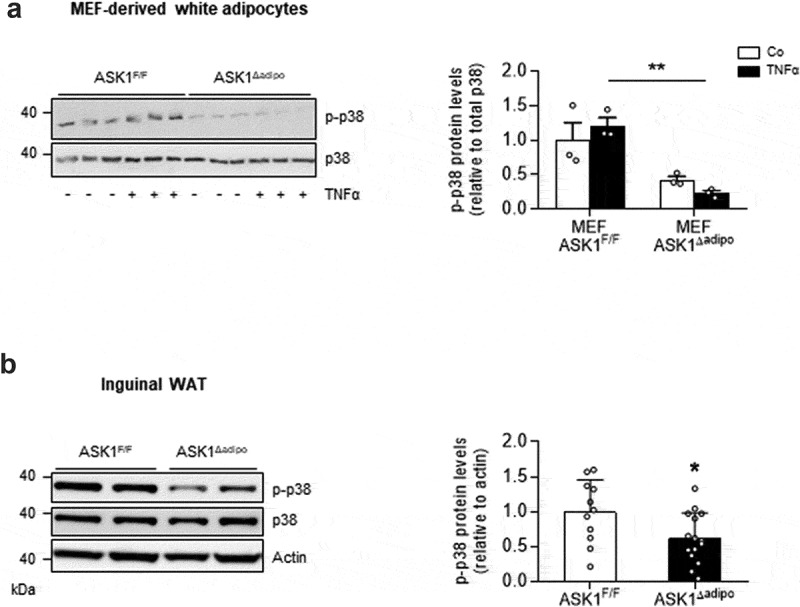
p38 MAPK is activated ASK1-dependently in adipocytes

To investigate whether adipose tissue-expressed p38 MAPK is activated ASK1-dependently, experiments in adipocytes derived from mouse embryonic fibroblasts (MEFs) isolated from ASK1^F/F^ and ASK1^Δadipo^ mice were performed. TNFα-induced phosphorylation of p38 MAPK was significantly blunted in adipocytes derived from ASK1-depleted adipocytes (Figure 1(a)), indicating ASK1-dependency of TNFα-induced p38 MAPK phosphorylation. Similarly, we previously showed that combined stimulation of TNFα and interleukin (IL)-1β induced phosphorylation of p38 in MEFs from ASK1^F/F^ but not from ASK1^Δadipo^ mice [15]. Next, we aimed to examine whether ASK1 depletion reduces phospho-p38 levels *in vivo*. As depicted in Figure 1(b), phospho-p38 protein levels were significantly reduced in inguinal WAT of HFD-fed ASK1^Δadipo^ compared to ASK1^F/F^ mice. In contrast, no difference in p38 MAPK phosphorylation was found in BAT of HFD-fed mice (1.00 ± 0.25 in ASK1^F/F^ mice vs. 1.05 ± 0.41 in ASK1^Δadipo^ mice, p = 0.92). Taken together, p38 MAPK is activated ASK1-dependently in white subcutaneous adipocytes.

(a) Western blot (left panel) and quantified protein levels (right panel) of phosphorylated and total p38 MAPK in MEF-derived adipocytes isolated from ASK1^F/F^ and ASK1^∆adipo^ mice treated with or without recombinant TNFα. (b) Western blot and quantified protein levels of phosphorylated and total p38 MAPK in inguinal WAT of 12-week HFD-fed ASK1^F/F^ (n = 11) and ASK1^∆adipo^ (n = 15) mice. Values are expressed as mean ± SEM. *p < 0.05 (*t*-test), **p < 0.01 (two-way ANOVA).

### Senescence markers are reduced in ingWAT of ASK1-deficient mice

**Figure 2.**
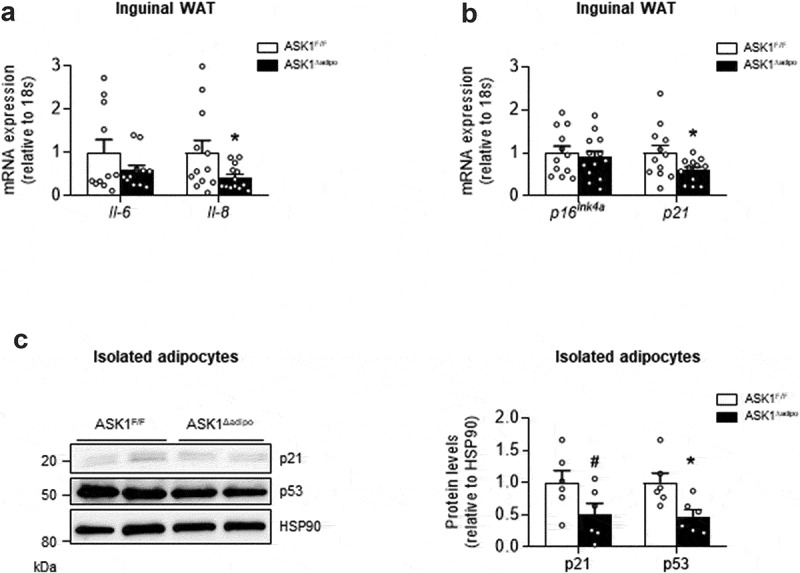
Senescence markers are reduced in ASK1-deficient mice

As activation of p38 MAPK induces cellular senescence [6], we aimed to examine whether reduced phospho-p38 MAPK protein levels in inguinal WAT of knockout mice were paralleled by blunted senescence. Of note, the pro-inflammatory cytokines IL-6 and IL-8 are important components of the senescence-associated secretory phenotype (SASP) of senescent cells [17]. In line with a role of ASK1 in adipocyte senescence, mRNA expression of the SASP cytokines *Il-6* and *Il-8* was reduced in inguinal WAT of HFD-fed ASK1^Δadipo^ mice (Figure 2(a)). Moreover, expression of the senescence marker *p21* was significantly reduced in knockout mice, whereas *p16^Ink4a^* mRNA levels were similar between the genotypes (Figure 2(b)). Overall, reduced expression of SASP cytokines as well as *p21* suggest blunted senescence in WAT of HFD knockout mice. However, these results do not allow to identify the source of ASK1-dependent regulation of senescence, i.e. whether senescence markers were reduced in adipocytes and/or in cells from the stromal vascular fraction of inguinal WAT, both relevant to WAT beiging [2]. We therefore determined p21 and p53 protein levels in isolated adipocytes of HFD-fed ASK1^Δadipo^ mice. As depicted in Figure 2(c), protein levels of both senescence markers were reduced by ~50% in knockout mice, indicating that ASK1 affects senescence status in adipocytes.

(a) mRNA expression of *Il-6* and *Il-8* in inguinal WAT of 12-week HFD-fed ASK1^F/F^ (n = 11–12) and ASK1^∆adipo^ (n = 12–13) mice. (b) mRNA expression of p16^Ink4α^ and *p21* in inguinal WAT of 12-week HFD-fed ASK1^F/F^ (n = 11–12) and ASK1^∆adipo^ (n = 12–13) mice. (c) Western blot and quantified protein levels of p21 and p53 in isolated adipocytes of 12-week HFD-fed ASK1^F/F^ (n = 6) and ASK1^∆adipo^ (n = 6) mice. Values are expressed as mean ± SEM. ^#^p = 0.08; *p < 0.05 (*t*-test).

### LPS induces senescence markers ASK1-dependently in subcutaneous white adipocytes

**Figure 3.**
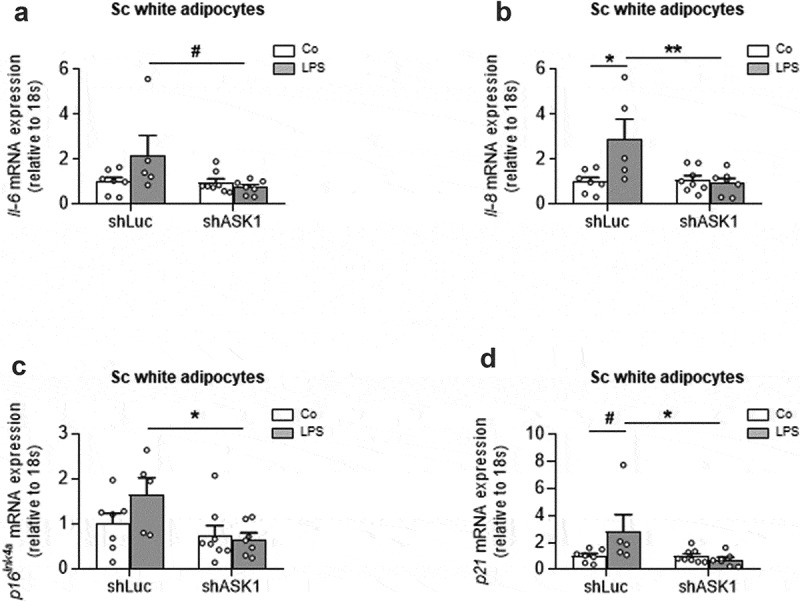
LPS induces senescence markers ASK1-dependently in subcutaneous white adipocytes

To further examine whether ASK1-depletion affects senescence in subcutaneous adipocytes, we utilized an *in vitro* approach, in which ASK1 was knocked down in adipocytes derived from murine subcutaneous (sc) white adipose tissue. ASK1 knock-down was achieved using a lentiviral construct expressing a short hairpin RNA (shRNA) against ASK1 (shASK1) leading to significantly reduced ASK1 expression [4]. Of note, LPS may not only activate ASK1 but it also induces senescence as well as the expression of SASP-cytokines such as IL-6 or IL-8 [18,19]. We therefore hypothesized that LPS-induced senescence is reduced in ASK1-depleted sc adipocytes. Indeed, ASK1 depletion reduced LPS-induced *Il-6 and IL-8* upregulation (Figure 3(a and b)), suggesting that ASK1 mediates to LPS-induced SASP in adipocytes. Moreover, LPS-induced expression of the senescence markers *p16^Ink4a^* and *p21* was blunted in ASK1-depleted cells (Figure 3(c and d)). These results suggest that ASK1 mediates LPS-induced senescence in subcutaneous adipocytes.

mRNA expression of *Il-6* (A), *Il-8* (B),  *p16^Ink4α^* (C) and *p21* (D) in subcutaneous adipocytes transfected with control shRNA lentivirus (shLuc) or shRNA lentivirus targeting ASK1 (shASK1) pre-treated with or without 100 ng/ml LPS for 24 hours followed by stimulation with 0.1 µM isoproterenol for 6 hours. n = 7 (shLuc control), n = 8 (shASK1 control), n = 5 (shLuc LPS) or n = 7 (shASK1 LPS). Values are expressed as mean ± SEM. ^#^p = 0.07 (for *p21*); ^#^p = 0.05 (for *Il-6*); *p < 0.05; **p < 0.01 (two-way ANOVA).

## Discussion

Increased senescence in adipose tissue may impact on local and systemic metabolism [7]. Accordingly, identification of factors regulating senescence in cells residing in WAT is of importance. Herein, we provide evidence that ASK1 contributes to stress-induced senescence in adipocytes. In fact, LPS-induced upregulation of senescence marker was reduced in ASK1-depleted subcutaneous adipocytes. Moreover, ASK1 contributed to LPS-induced expression of the SASP-cytokines *Il-6* and *Il-8*. In vivo, increased IL-6 and IL-8 levels not only act autocrinally but they may also induce senescence in other cells residing in WAT [7,20]. In line with a role of ASK1 in the adipocyte senescence, *p21* as well as the SASP marker *Il-8* were significantly reduced in inguinal fat of HFD-fed ASK1^Δadipo^ mice. Of note, plasma IL-8 levels were similar between HFD-fed ASK1^F/F^ and ASK1^Δadipo^ mice [4], indicating that reduced *Il-8* expression in inguinal WAT did not affect its circulating levels. In obesity, increased adipocyte senescence characterized by elevated expression of *p16^Ink4a^* and *p21* was associated with elevated body weight and impaired glucose tolerance in HFD-fed mice [21]. Moreover, adipocyte-specific depletion of the senescence modulator p53 ameliorated HFD-induced glucose intolerance in mice [22]. These data suggest a role for adipocyte senescence in HFD-induced body weight gain and/or glucose intolerance. Herein, we revealed that p21 and p53 levels were reduced in adipocytes/WAT of HFD-fed ASK1^Δadipo^ mice. Accordingly, reduced senescence in subcutaneous WAT may have contributed to reduced body weight gain and/or ameliorated glucose metabolism in these mice [4]. Of note, HFD-feeding increases senescence in WAT [22]. In addition, we previously reported significantly elevated total and phosphorylated ASK1 protein levels in inguinal WAT of HFD-fed mice, indicating increased ASK1 activation [4]. Thus, increased ASK1 activation may contribute to elevated senescence in adipocytes of HFD-fed mice.

As outlined above, p38 MAPK contributes to the induction of senescence in adipocyte progenitors [22]. In support of a role of this stress kinase in senescence of mature adipocytes, we show herein that p38 MAPK is activated ASK1-dependently in the latter. In addition, decreased expression of senescence marker in inguinal WAT of knockout mice were associated with reduced phopho-p38 protein levels. In parallel, UCP1 protein levels were significantly elevated [4], indicating that p38 MAPK may negatively affect UCP1 levels in subcutaneous WAT. In contrast, protein levels of UCP1 [4] and phospho-p38 MAPK were similar in BAT of both genotypes. In the latter tissue, p38 MAPK was reported to positively control UCP1 expression [23]. Moreover, experiments in cultured white adipocytes revealed that atrial natriuretic peptide (ANP) and irisin-induced upregulation of UCP1 protein levels is blocked using a p38 MAPK inhibitor [24,25]. Similarly, irisin-induced UCP1 protein levels p38 MAPK-dependently in human white adipocytes but not in human perirenal BAT [26]. In contrast, lack of the p38 MAPK upstream activator MAPK 6 (MKK6) elevated browning of WAT [27] and adipocyte-specific depletion of the isoform p38α increased UCP1 protein levels in inguinal WAT [28]. Since in human obesity over-activated MKK6-p38 MAPK seemed downstream of up-regulated ASK1, these data suggest a potential mechanism for obesity-related adipose tissue ‘whitening’ – i.e. the adaptation to a more energy accumulating and less dissipating adipose tissue phenotype. Overall, the role of p38 MAPK in the regulation of WAT browning remains controversial and needs further clarification.

As non-dividing cells, mature adipocyte senescence may be controlled differently than in dividing cells. Of note, we previously demonstrated in human adipose tissue that ASK1 is transcriptionally up-regulated in obesity by binding of the transcription factor E2F1 to its promoter. E2F1 is a positive regulator of cell cycle, and is up-regulated in various aggressive (rapidly-dividing) cancer cells [15]. In such cells, E2F1 also acts to repress senescence by regulating various senescence inhibitors after their promoters become available for E2F1 binding [29]. Conversely, in the non-dividing adipocytes in obesity, the repertoire of E2F1 binding sites in the genome may be altered, where it may activate non-classical E2F1 targets such as autophagy genes and ASK1. Thus, E2F1-ASK1-p38 MAPK may act in a senescence-promoting process in mature white adipocytes in obesity.

In conclusion, our study identifies a previously unknown role for ASK1 in the induction of stress-induced senescence in white adipocytes. Accordingly, blocking ASK1 in adipocytes may be an attractive approach to reduce body weight and/or to improve glucose metabolism.
